# Immune depletion of the methylated phenotype of colon cancer is closely related to resistance to immune checkpoint inhibitors

**DOI:** 10.3389/fimmu.2022.983636

**Published:** 2022-09-08

**Authors:** Chengqian Zhong, Tingjiang Xie, Long Chen, Xuejing Zhong, Xinjing Li, Xiumei Cai, Kaihong Chen, Shiqian Lan

**Affiliations:** ^1^ Department of Digestive Endoscopy center, Longyan First Affiliated Hospital of Fujian Medical University, Longyan, China; ^2^ Department of Gastrointestinal Surgery, Longyan First Affiliated Hospital of Fujian Medical University, Longyan, China; ^3^ Department of Science and Education, Longyan First Affiliated Hospital of Fujian Medical University, Longyan, China; ^4^ Department of Pathology, Longyan First Affiliated Hospital of Fujian Medical University, Longyan, China; ^5^ Department of Cardiology, Longyan First Affiliated Hospital of Fujian Medical University, Longyan, China

**Keywords:** colorectal cancer, molecular subtype, machine learning, methylation, immunotherapy

## Abstract

**Background:**

Molecular typing based on single omics data has its limitations and requires effective integration of multiple omics data for tumor typing of colorectal cancer (CRC).

**Methods:**

Transcriptome expression, DNA methylation, somatic mutation, clinicopathological information, and copy number variation were retrieved from TCGA, UCSC Xena, cBioPortal, FireBrowse, or GEO. After pre-processing and calculating the clustering prediction index (CPI) with gap statistics, integrative clustering analysis was conducted *via* MOVICS. The tumor microenvironment (TME) was deconvolved using several algorithms such as GSVA, MCPcounter, ESTIMATE, and PCA. The metabolism-relevant pathways were extracted through ssGSEA. Differential analysis was based on limma and enrichment analysis was carried out by Enrichr. DNA methylation and transcriptome expression were integrated *via* ELMER. Finally, nearest template or hemotherapeutic sensitivity prediction was conducted using NTP or pRRophetic.

**Results:**

Three molecular subtypes (CS1, CS2, and CS3) were recognized by integrating transcriptome, DNA methylation, and driver mutations. CRC patients in CS3 had the most favorable prognosis. A total of 90 differentially mutated genes among the three CSs were obtained, and CS3 displayed the highest tumor mutation burden (TMB), while significant instability across the entire chromosome was observed in the CS2 group. A total of 30 upregulated mRNAs served as classifiers were identified and the similar diversity in clinical outcomes of CS3 was validated in four external datasets. The heterogeneity in the TME and metabolism-related pathways were also observed in the three CSs. Furthermore, we found CS2 tended to loss methylations while CS3 tended to gain methylations. Univariate and multivariate Cox regression revealed that the subtypes were independent prognostic factors. For the drug sensitivity analysis, we found patients in CS2 were more sensitive to ABT.263, NSC.87877, BIRB.0796, and PAC.1. By Integrating with the DNA mutation and RNA expression in CS3, we identified that SOX9, a specific marker of CS3, was higher in the tumor than tumor adjacent by IHC in the in-house cohort and public cohort.

**Conclusion:**

The molecular subtypes based on integrated multi-omics uncovered new insights into the prognosis, mechanisms, and clinical therapeutic targets for CRC.

## Introduction

Colorectal cancer (CRC) is the third most common malignant tumor in the world and the fourth major cause of cancer death (1). The diagnosis of CRC is often in the middle and late stages with poor prognosis, and distant metastasis is the main cause of death in colorectal cancer patients. With the continuous improvement in medical level, comprehensive treatment measures such as surgery, radiotherapy, and chemotherapy, targeted therapy, and immunotherapy have improved the overall survival (OS) of patients with CRC, but their overall efficacy is still poor, and the 5-year survival rate of patients with metastatic CRC is only about 14% (1). Therefore, how to effectively evaluate the prognosis of different CRC patients is an urgent problem to be solved.

At present, the most widely used prognostic staging system for CRC is the TNM (Tumor, Node, and Metastasis) staging system, which is easy to observe from clinical information and is the benchmark for the establishment of clinical treatment plans for patients. However, the TNM staging system mainly relies on expert opinions, and the features used are relatively single. The abnormal phenomenon of the TNM staging system in CRC (the prognosis of patients at stage IIB/C is significantly worse than that of patients at stage III A) results in its limited ability of personalized and accurate clinical decision (2). At the same time, as a population-based system, the TNM staging system has been questioned about its application to individual patients (3). The latest eighth edition of the TNM staging system included biomarkers as new prognostic factors in some cancer staging (3). Therefore, it is necessary to introduce new prognostic factors to the existing TNM staging system in order to more accurately assess the prognosis of patients and formulate treatment plans.

Cancer is a complex disease with high heterogeneity, even patients with the same histopathological classification will have different gene mutations (4). Hence, personalized prevention, diagnosis, and treatment should be done according to the clinical and omics characteristics of different patients (5). For CRC, microsatellite instability (MSI), DNA mismatch repair (MMR), and the results of molecular tests such as RAS mutation and BRAF VE6000 are used to determine the prognosis (3, 6). It is possible to combine clinical and omics information for more personalized prognostic analysis of cancer, but it is difficult for a single omics data to fully account for all factors in a complex disease such as cancer, making it difficult for researchers to derive data from millions of single-nucleotide variations (SNV) to find the key gene that actually causes the disease (7). In recent years, more and more researchers have carried out integrated analysis of various omics data and obtained certain results (8, 9). However, most prognostic studies of CRC are limited to one set of omics, such as gene expression (10) or DNA methylation (11), and few studies that consider multiple omics data have failed to effectively combine multiple omics data with clinical data (12). Therefore, how to integrate clinical data and omics data and apply them to the prognosis of CRC is of great significance.

The Cancer Genome Atlas (TCGA) is a platform that integrates clinical data, survival information, and multiple omics data for 33 cancers. Through the integration and analysis of multiple omics, cancer subtype classification, biomarker discovery, and survival prognosis analysis can be carried out (13–15). Herein, using data from TCGA and other public databases, we developed a classifier based on multi-omics integration for the prognosis prediction of CRC for the first time. We evaluated the differences in genomic heterogeneity, transcriptome biomarkers, TME landscape, metabolism-related pathways, epigenetic regulation, and chemotherapeutic drug sensitivity among the molecular subtypes of CRC. Multivariate Cox regression analysis confirmed the independent prognostic value of our subtype system. In summary, the molecular subtypes based on integrated multi-omics uncovered new insights into the prognosis, mechanisms, and clinical therapeutic targets for patients with CRC.

## Materials and methods

### Study population

Molecular data of patients diagnosed with CRC were retrieved from TCGA (13). Transcriptome expression profiles of the TCGA-COAD (colon adenocarcinoma) and TCGA-READ (rectum adenocarcinoma) projects quantified by the number of fragments per kilobase million (FPKM) were downloaded from the UCSC Xena (https://xenabrowser.net/), including 616 fresh-frozen samples with primary malignancy and 51 adjacent normal samples. The DNA methylation profile quantified by Illumina HumanMethylation 450K-array platform was downloaded from the UCSC Xena (https://xenabrowser.net/) under the projects of TCGA-COAD and TCGA-READ, respectively, including a total of 387 primary colorectal tumour samples and 45 adjacent normal samples. Somatic mutation data, patients’ clinicopathological information, and survival data were retrieved from cBioPortal (http://www.cbioportal.org/datasets) (16). Copy number variation (CNV) data was collected from FireBrowse (http://firebrowse.org/) (17). For the purpose of multi-omics integrative clustering, 306 primary colorectal tumour samples with available transcriptome expression, DNA methylation, and somatic mutation profiles were identified for this study. Another four independent cohorts downloaded from GEO, including GSE14333 (18), GSE17538 (19), GSE38832 (20), and GSE39582 (21), comprised of a total of 1,159 CRCs with gene expression matrix and corresponding clinicopathological information. Of these external validation cohorts, gene expression matrices were profiled by Affymetrix Human Genome U133 Plus 2.0 Array. The Robust Multichip Average (RMA) algorithm was used for background correction and normalization (22).

### Data pre-processing for gene expression and DNA methylation profiles

For the FPKM data of high-throughput sequencing from TCGA, Ensembl IDs for mRNAs were transformed to gene symbols by GENCODE 22. The FPKM values were transferred into transcripts per kilobase million (TPM) values, which showed more similarity to those derived from microarray and more comparable between samples (23). For microarray data retrieved from GEO database, we performed RMA normalization and processing using default settings for background correction and normalization by R package affy (24). Affymetrix probe ID was annotated with gene symbols according to the GPL570 platform. For multiple probes that mapped to one gene, mean value of expression was considered. The potential cross-dataset batch effect was removed under an empirical Bayes framework, namely, ComBat, by the R package sva (25), and the batch effect was further investigated using principal component analysis (PCA) for transcriptome profiles. For DNA methylation, we performed logit transforms β-values before ComBat adjustment and then computed the reverse logit transformation following the ComBat adjustment (26). Subsequently, we used R package ChAMP to comprehensively filter the methylation matrix. To be specific, probes with detection *P* value > 0.01, probes with <3 beads in at least 5% of samples per probe, all non-CpG probes, all SNP-related probes, all multi-hit probes, and probes located on sex chromosomes were removed in the first place (26, 27).

### Integrative clustering based on multi-omics profiles

To perform integrative clustering analysis, we processed the TCGA multi-omics data sets to form three data matrices with columns corresponding to the common samples (n = 306) and rows corresponding to the omics features. The transcriptome expression profile was first log_2_ transformed. For the methylation data, we extracted probes located in promoter CpG islands, and for genes having more than one probe mapping to its promoter, the median β value was considered to identify 10,263 methylated genes. For the mutation matrix, a gene was considered mutated (entry of 1) if it contained at least one type of the following nonsynonymous variations: frameshift deletion/insertion, in-frame deletion/insertion, missense/nonsense/nonstop mutation, splice site or translation start site mutation; otherwise, 0 was used to designate wild-type status. To better fit the model and accelerate the clustering efficiency, features with flat values were removed. Specifically, we used the top 1,500 most variable mRNAs, and methylation genes according to the median absolute deviation. Additionally, 20 genes that were previously identified as driver mutations for colorectal carcinoma were selected for cancer subtyping (28). To find an optimal clustering number, we calculated the clustering prediction index (CPI) and gap statistics using R package MOVICS (29). Consequently, integrative clustering of the TCGA cohort was conducted by R package MOVICS using a Bayesian latent variable model (29, 30).

### Deconvolution of tumour microenvironment

To estimate the cell abundance of TME, we retrieved from the previous study a compendium of microenvironment genes related to specific microenvironment cell subsets, which consisted of 364 genes representing 24 microenvironment cell types (31, 32). We then used gene set variation analysis (GSVA) on these gene sets to generate enrichment scores for each cell using the R package GSVA (33). Additionally, quantification of the absolute abundance of eight immune and two stromal cell populations in heterogeneous tissues from transcriptomic data was conducted by the R package MCPcounter (34). The presence of infiltrating immune/stromal cells in the tumour tissue was estimated by the R package ESTIMATE (35). Additionally, the individual DNA methylation of tumour-infiltrating lymphocyte (MeTIL) score in the TCGA cohort was calculated using PCA according to the protocols described in the literature (36).

### Single sample enrichment for metabolism-relevant pathways

The 115 metabolism-relevant gene signatures were achieved from previously published study (37), and were quantified by using single-sample GSEA (ssGSEA) approach through R package GSVA (38). Specifically, we extracted three main categories of these metabolism-relevant pathways, including carbohydrate metabolism, amino acid metabolism, and lipid metabolism.

### Differential analysis and functional enrichment

Differential expression analyses were conducted using the R package “*limma*” (39). Gene set enrichment analysis (GSEA) was performed based on pre-ranked gene list according to the descending ordered log_2_FoldChange value derived from differential expression analysis; we then leveraged R package clusterProfiler to determine functional enrichment based on Hallmark gene set background that was retrieved from Molecular Signatures Database (MSigDB) (40, 41). The differentially methylated probes (DMPs) were obtained by R package ChAMP (26). Specifically, we considered probe to have significantly gained methylation if its corresponding mean β-value was greater than 0.3 in the specific subtype but less than 0.2 in the reference subtype with *P*<0.05 and FDR<0.05; vice versa for probes that significantly lost methylation. Gene-list based enrichment analysis was conducted by an integrative and collaborative website tool (Enrichr; https://maayanlab.cloud/Enrichr/) (42).

### Cancer subtype characterization and visualization

As previously developed R package MOVICS provides powerful functions to comprehensively characterize cancer subtypes and create feature rich customizable visualizations with minimal effort, we therefore characterized the identified colorectal subtypes from multiple aspects, including survival rate, mutational frequency, fraction of copy number-altered genome (FGA), and clinical characteristics. All parameters were set to default values (29).

### Integrative analysis of DNA methylation and transcriptome expression

We used R package ELMER to investigate the crosstalk between DNA methylation and transcriptome expression under an integrative analytic pipeline (43). For probes that are located in promoters, we identified putative genes that were significantly downregulated due to the hypermethylation of promoter probes. Next, the closest 20 upstream and downstream genes were collected for each probe, and for each candidate probe-gene pair, the Mann-Whitney U test was harnessed to test the null hypothesis that overall gene expression in the specific group was less than or equal to that in the reference group. For probes that are located in enhancers (distal probes that are at least 2Kb away from transcription start site on human chromosomes), hypomethylated enhancer mode with overexpressed gene expression pattern was investigated accordingly.

### Nearest template prediction

Gene-expression signature-based classification was conducted using NTP algorithm, which provided a convenient model-free approach to make category prediction at single-sample level using only a list of signature genes and a test dataset, which was flexible and beneficial in external cohort application (44, 45).

### Analysis of regulons

Transcriptional regulatory networks (regulons) were constructed for 71 candidate regulators associated with cancerous chromatin remodelling (46). As described in the previous study (31), potential associations between a regulator and all possible target genes were revealed by mutual information and Spearman’s correlation, and associations were dropped *via* permutation analysis if the corresponding FDR was greater than 0.00001. Unstable associations were also eliminated through bootstrapping (1,000 re-samplings, consensus bootstrap>95%), and the weakest associations were removed by data processing inequality (DPI) filtering embedded in the R package RTN (47). Regulon activity scores for all samples were calculated by two-tailed GSEA.

### Therapeutic response analysis

We employed R package pRRophetic to predict the chemotherapeutic sensitivity for each colorectal sample using the parameters by default (48, 49). For immunotherapy, we retrieved a published data set consisting of 47 patients with melanoma who responded to anti-CTLA4 or anti-PD1 blockades (50), and then harnessed subclass mapping to predict the clinical response to immune checkpoint blockade (51).

### Immunohistochemical staining

The 50 pairs of CRC tumor and adjacent normal tissue Microarray (D216Re01) were purchased from Xi’an bioaitech Co., Ltd (Xi’an, China). Immunohistochemical staining was performed on normal and the paired tumor tissue slides. The slides were incubated with rabbit polyclonalanti-SOX9 (EPR14335, 1:2000); antibodies at 4℃ overnight. SOX9 expression was evaluated by using a system considering the staining intensity (0 means negative 1 means weak; 2 means moderate; and 3 means strong) and the percentage of positively stained cells (<5%=05% to <25%=1, 25% to 50%=2, >50 to <75%=3, >75%=4). The final score was calculated by multiplying the extent score by the intensity score.

### Statistical analyses

All statistical analyses were performed by R (Version 4.0.2). We used Fisher’s exact test for categorical data, Kruskal–Wallis one-way analysis of variance for continuous data, a log-rank test for Kaplan-Meier curve, and Cox regression for hazard ratio. For all comparisons, a two-sided *P* < 0.05 was considered statistically significant.

## Results

### Multi-omics integrative molecular subtype of colorectal cancer

**Figure 1:**
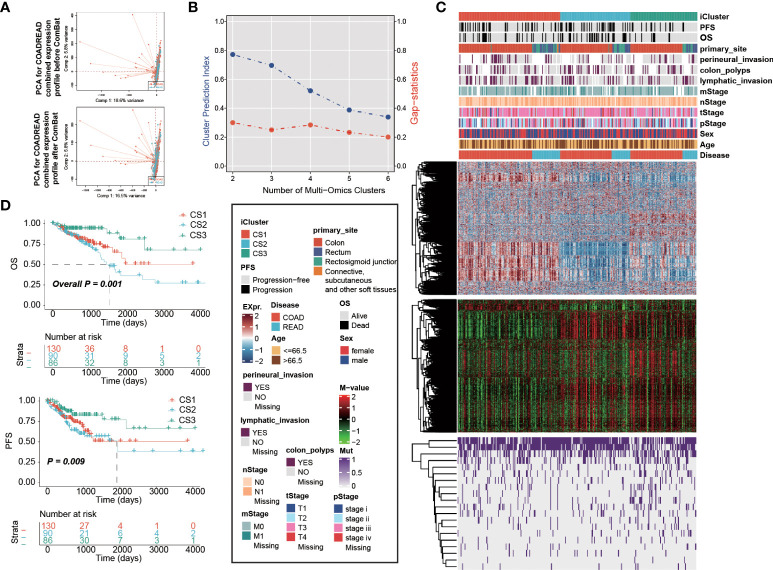
Multi-omics integrative molecular subtype of colorectal cancer. Principal component analysis to investigate the potential batch effect between TCGA-COAD and TCGA-READ. **(A)** before and after Combat. **(B)** Identification of optimal clustering number by calculating CPI and Gaps-statistics. **(C)** Comprehensive heatmap showing the molecular landscape of three cancer subtypes of colorectal carcinoma using integrative clustering. Kaplan-Meier curves of **(D)** OS and PFS with log-rank test for 306 patients with colorectal cancer according to the current molecular classification.

We combined expression profiles of TCGA-COAD and TCGA-READ, and further removed the potential batch effect (**Figure 1A**). We determined the optimal cluster number of three taking into account two clustering statistics and previous molecular classifications (**Figure 1B**). Subsequently, integrative clustering identified three robust cancer subtypes (CSs), which were characterized by distinct molecular patterns across transcriptome mRNA expression, DNA methylation and colorectal cancerous driver mutations (**Figure 1C**). Of note, these classifications were not associated with major clinical features (all *P* > 0.05; **Supplementary Table S1**); our classification system was tightly associated with overall survival rate (OS; *P* = 0.001; **Figure 1D**) and progression-free survival rate (PFS; *P* = 0.009);. Generally, CS3 showed the most favourable prognosis among three clusters.

### Genomic heterogeneity of colorectal cancer subtype

**Figure 2:**
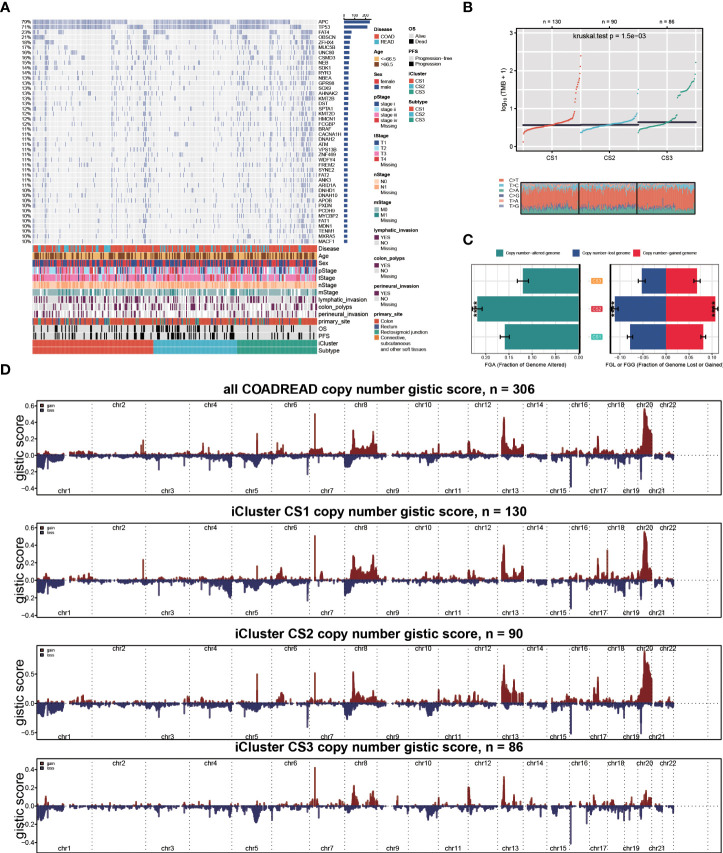
Genomic heterogeneity of colorectal cancer subtype. **(A)** OncoPrint showing the distribution of genes that were differentially mutated between three cancer subtypes. **(B)** Distribution of TMB and TiTv (transition to transversion) between two epigenetic phenotypes. **(C)** Barplot showing the distribution of FGA and fraction genome gain/loss (FGA/FGG). Bar charts are presented as the mean ± standard error of the mean. **(D)**three types distinguishing composite copy number. ****p<0.0001.

To investigate the genomic heterogeneity of these molecular subtypes further, we investigate the differentially mutated genes among our classifications, leading to a total of 90 genes (FDR < 0.05 and mutational frequency > 10%; **Figure 2A**). Among these 90 genes, 11 genes were previously identified as driver mutations in colorectal cancer, including *PIK3CA*, *APC*, *BRAF*, *KRAS*, *TP53*, *FBXW7*, *AMER1*, *TCF7L2*, *SOX9*, *ARID1A*, and *SMAD4* (**Supplementary Table S2**). Additionally, we found that CS3 showed a significantly higher tumour mutation burden (TMB, *P* = 0.002; **Figure 2B**) than the other two subtypes. We then investigated chromosomal instability by calculating the FGA scores and found that CS2 had significant instability across the entire chromosome as compared to the other two subtypes with significantly higher copy number loss or gain (*P* < 0.001; **Figure 2C**). We showed three types distinguishing composite copy number profiles: gistic score (**Figure 2D**), and percentage/frequency.

### Identification of transcriptome biomarkers for colorectal cancer subtype

**Figure 3:**
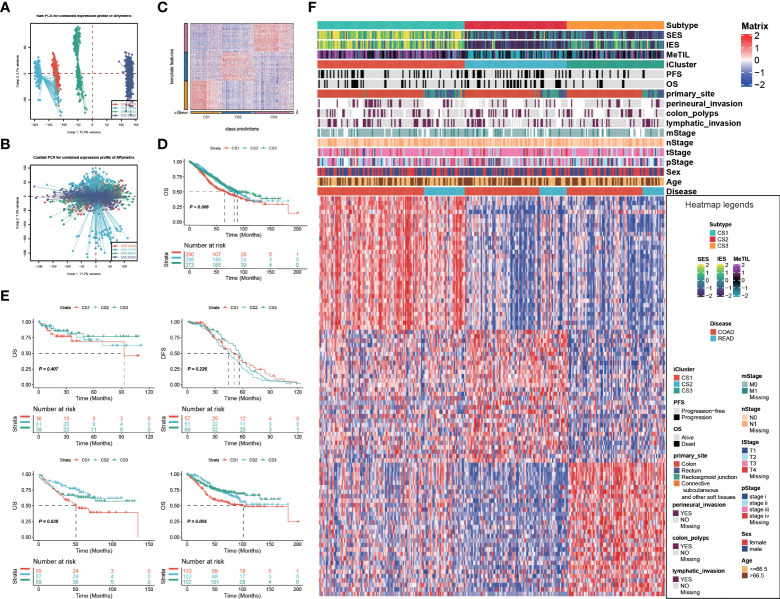
Identification of transcriptome biomarkers for colorectal cancer subtype. Principal component analysis to investigate the potential batch effect among four GEO datasets **(A)** before and **(B)** after Combat. **(C)** Heatmap showing the transcriptome expression pattern of the 120-gene signature in nearest template predicted cancer subtype of GEO cohort. **(D)** Kaplan-Meier curves of OS with log-rank test for 961 patients with colorectal cancer according to the eligible predicted classification. **(E)** KM of os using ntp in GEO **(F)** Heatmap showing the transcriptome expression pattern of the 90-gene signature (30 uniquely significantly upregulated genes in each cancer subtype) in TCGA cohort.

Given that transcriptome-level data were the most commonly used molecular profiles in cancer research, we identified 30 mRNAs with uniquely and significantly upregulated expression as classifiers for each subtype in the TCGA cohort, and a 90-gene signature was generated (**Figure 3F**; **Supplementary Table S3**). To test the reproducibility of our identified colorectal molecular subtypes, we combined four external datasets as GEO cohort of which expression profiles were measured by microarray platform; batch effect across different datasets were removed (**Figures 3A** **,** **B**). We then predict the identified molecular subtypes in the GEO cohort (n = 1,159) using NTP algorithm, which classified each sample in the GEO cohort as one of the identified CS (**Figure 3C**). Of note, a total of 961 cases of GEO cohort were predicted with confidence (FDR < 0.05) and those cases were used for the downstream analyses. Likewise, CS3 presented with the most favourable clinical outcome out of the three subtypes (*P* = 0.008; **Figures 3D** **,** **E**).

### Delineation of metabolism-related pathways in colorectal cancer subtype

**Figure 4:**
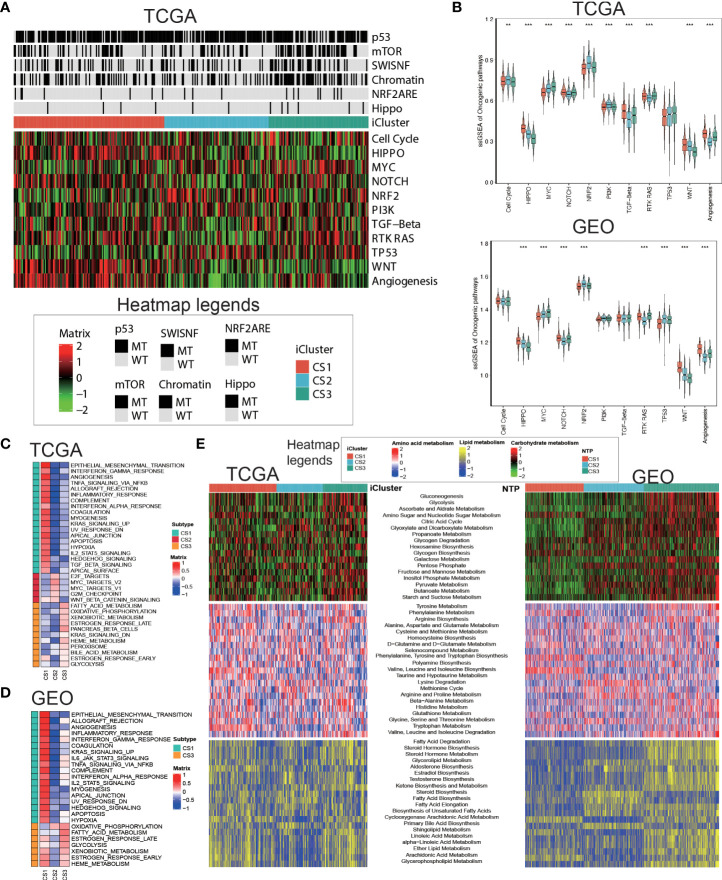
Delineation of metabolism-related pathways in colorectal cancer subtype. **(A)** Oncogenic heatmap with cancer associated mutations in tcga coadread. **(B)** Boxplot for oncogenetic pathways in iclusters of tcga coadread. **(C)** Upregulated hallmark pathway heatmap in tcga_using_upregulated_pathways. **(D)** Upregulated hallmark pathway heatmap in geo_using_upregulated_pathways. **(E)** Heatmap showing transcriptome enrichment score of three metabolic categories in TCGA and GEO cohorts. **p < 0.01; ***p < 0.001.

Oncogenic heatmap with cancer associated mutations in tcga coadread (**Figure 4A**). Boxplot for oncogenetic pathways in iclusters of tcga coadread(**Figure 4B**). Similarly, GSEA is run for each subtype based on its corresponding DEA result to identify subtype-specific functional pathways (**Figures 4C** **,** **D**). Since Metabolic pathways regulate colorectal cancer initiation and progression, we further explored whether distinct subtypes had different metabolic characteristics in both TCGA and GEO cohort (**Figure 4E**). Of note, we found global dysfunction of metabolism-related pathways among three molecular subtypes, and generally CS3 showed relatively higher enrichment level of carbohydrate, amino acid, and lipid metabolism-relevant pathways, which may suggest that these colorectal cancers preserved the default metabolic program of normal colon and rectum, leading to a generally good clinical outcome.

### Tumour microenvironment landscape of colorectal cancer subtype

**Figure 5:**
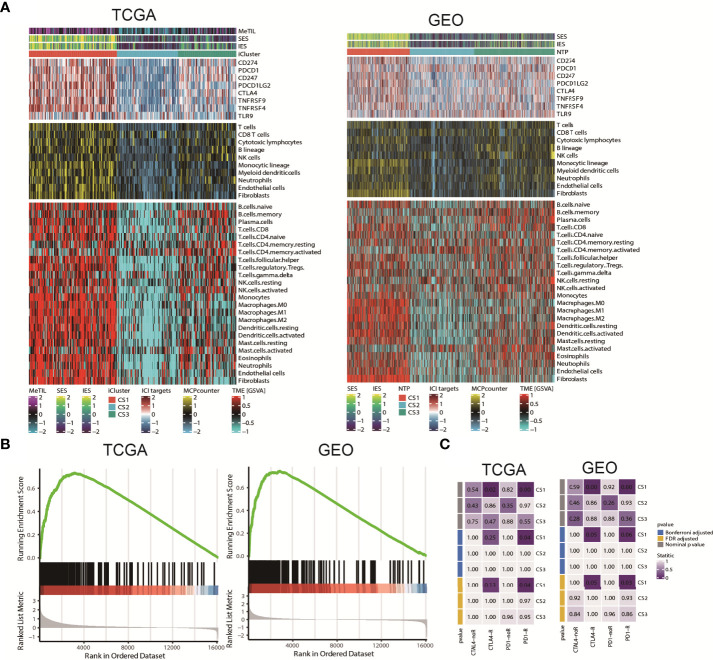
Tumour microenvironment landscape of colorectal cancer subtype. **(A)** Heatmap showing the immune profile in the TCGA and GEO cohort, with the top panel showing the expression of genes involved in immune checkpoint targets, the middle panel showing the enrichment level of 10 microenvironment cell types using MCPcounter approach, and the bottom panel showing the 24 microenvironment cells using GSVA approach; DNA methylation of tumour-infiltrating lymphocytes (MeTILs) were annotated at the top of the heatmap. The immune enrichment score and stromal enrichment score were annotated at the top of the heatmap. **(B)** GSEA plot showing activation of interferon-γ hallmark pathway. **(C)** Subclass analysis manifested that CS1 subtypes could be more sensitive to the immune checkpoint inhibitors.

Since cancer immunity plays a critical role in tumour progression, we suspected that the tumour microenvironment may vary a lot among these molecular subtypes. Since cancer immunity plays a critical role in tumour progression, we suspected that the tumour microenvironment may vary a lot among these molecular subtypes. Therefore, we investigated the specific immune cell infiltration status of samples in the TCGA cohort. To be specific, we quantified the infiltration levels of several microenvironment cell types using different approach, and surveyed the colorectal samples for the expression of genes representing immune checkpoint targets. The analysis of gene expression signatures suggested that CS1 was highly immune-infiltrated, CS3 showed relatively higher immunocyte infiltration, while CS2 was generally immune-depleted (**Figure 5A**). This finding may converge to the poor overall survival of CS2 versus other molecular subtypes. Compared to the other subtypes, CS1 had relatively higher expression of several genes that represent potential targets for immunotherapy, including CD274 (PDL1), PDCD1 (PD1), CD247 (CD3), PDCD1LG2 (PDL2), CTLA4 (CD152), TNFRSF9 (CD137), TNFRSF4 (CD134) and TLR9 (Sup_S2). Interestingly, CS1 enriched for B cell, CD8 T cells but may lack CD4 memory activated cells (Sup_S2); previous study showed the ratio of CD4/CD8 may play prognostic role in several cancer subtypes (52, 53). Additionally, we found that interferon-γ pathway was significantly activated in CS1 (FDR < 0.001; **Figure 5B**), which made us hypothesized that CS1 may be beneficial from immune checkpoint inhibitors. In this manner, we performed subclass mapping of TCGA cohort and revealed that only the CS1 showed high transcriptome-level similarity to a group of patients with melanoma who responded to anti-CTLA4 or anti-PD1 blockades (*P* < 0.05, adjusted *P* ≤0.25; **Figure 5C**), which indicated that the current classification may be useful to identify ideal candidates of patients with colorectal cancer for immunotherapy. The tumour microenvironment landscape was generally validated in GEO cohort. Consistently, CS1 in GEO cohort significantly activated interferon-γ pathway, and showed higher likelihood of responding to immune checkpoint inhibitors.

### Epigenetic regulation in colorectal cancer subtype

**Figure 6:**
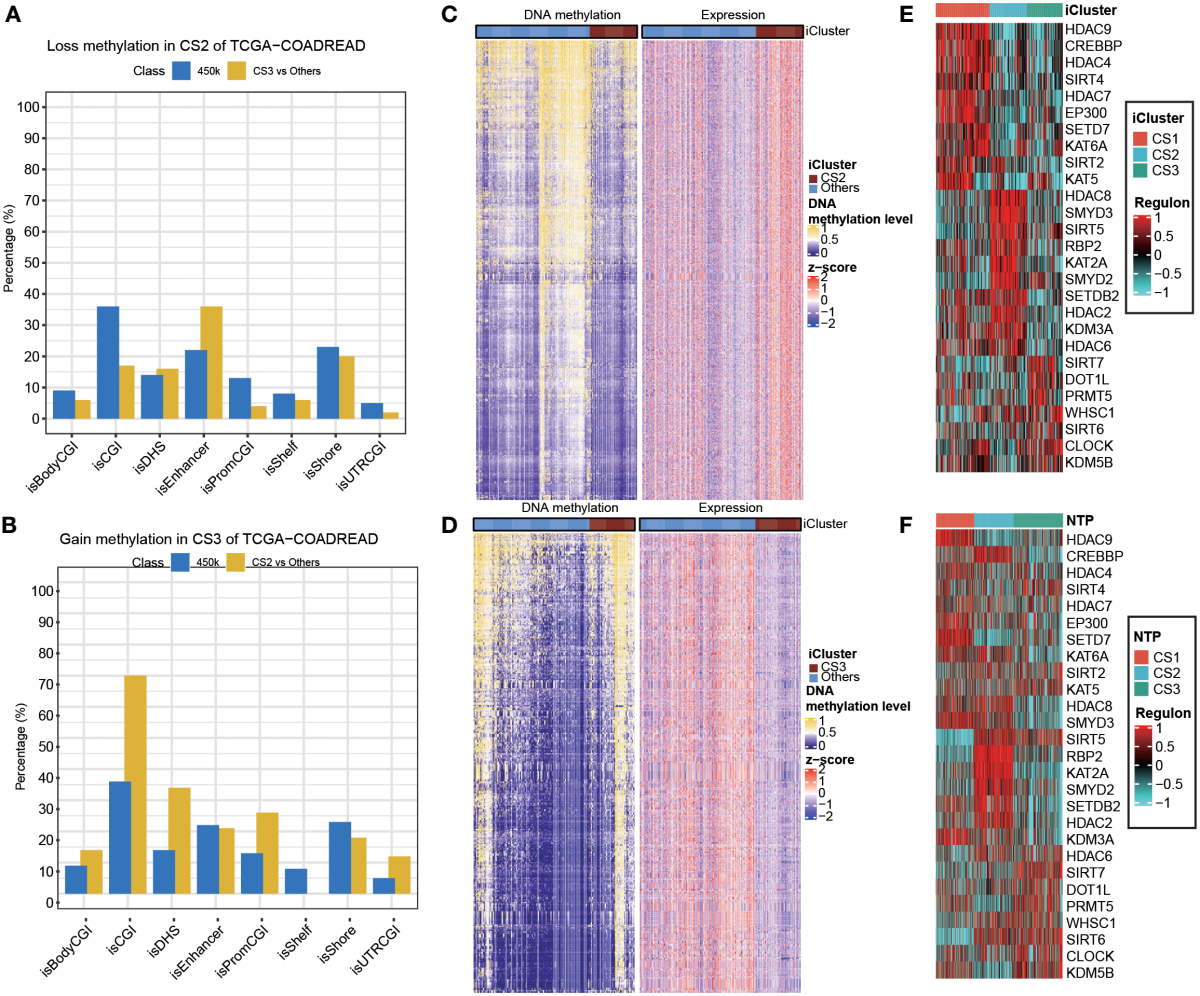
Epigenetic regulation in colorectal cancer subtype. Heatmap showing activity of regulon relevant to potential regulators associated with chromatin remodelling in both **(A)** TCGA and **(B)** GEO cohorts. Heatmap showing the association between DNA methylation and gene expression, presenting with **(C)** an epigenetic activation pattern in CS2 and **(D)** an epigenetically silencing pattern in CS3 of TCGA cohort. Barplots showing the region-specific distribution of DMPs comparing to the Illumina 450karray background for the **(E)** CS2 and **(F)** CS3 molecular classification in TCGA cohort.

Given the different transcription profiles among the three CRC subtypes, we then asked if this could mirror the epigenetic aspect. To this end, we identified differentially methylated probes for each subtype, and we found CS2 tended to loss methylations (n = 240) as compared to other subtypes (**Supplementary Table S4**). Notably, these probes losing DNA methylation were significantly enriched in enhancers compared to the 450K array background (*P*<0.001; **Figure 6A**). As to CS3, we found this subtype tended to gain methylations (n=249) compared to other subtypes (**Supplementary Table S5**), and those probes gaining methylation significantly enriched in promoter CpG islands (*P*<0.001; **Figure 6B**). To further investigate the crosstalk between epigenetic DNA methylation and transcriptome expression, we performed integrative analysis combining both gene expression and DNA methylation profiles using ELMER pipeline. First, for CS2, we identified distal probes that are 2Kb away from the transcription start site of the human chromosome, and performed differential methylation analysis at probe level to identify probes with difference of β-value greater than 0.1 (FDR<0.05) in CS2 compared to other subtypes, ending up with a total of 3,683 distal probes/enhancers (**Supplementary Table S6**). Next, ELMER searched for the nearby 20 genes corresponding to these probes, and further predicted enhancer-gene linkages using associations between DNA methylation at enhancers and expression of 20 nearby genes of the CpG sites; such analysis identified a total of 2,533 pairs corresponding to 1,003 genes (**Figure 6C**; **Supplementary Table S7**). To understand the biologic relevance of these genes that were epigenetically activated, we harnessed Enrichr and found that these genes were significantly enriched in MYC Hallmark pathways (*P* =0.006, FDR=0.24; **Supplementary Table S8**). Previous study demonstrated that MYC oncogene was associated the suppression in tumour immunity (54), which suggest that the downregulation of MYC oncogenic pathway may contribute shaping the immune-depleted tumour microenvironment of CS2. Using the similar strategy, we investigated CS3, but we searched for promoter-gene pairs that showed epigenetically silencing mode. In this manner, ELMER identified a total of 1,063 promoters that gained methylation in CS3 versus other subtypes (**Supplementary Table S9**), and a total of 3,212 promoter-gene pairs were identified to be epigenetically silenced in CS3 (**Figure 6D**; **Supplementary Table S10**). Enrichr revealed that these genes are significantly enriched in epithelia-mesenchymal transition (EMT) hallmark pathway (*P*<0.001, FDR<0.001; **Supplementary Table S11**). Down-regulation of EMT may decrease tumour-initiating and metastatic potential of cancer cells (55), which lead to good prognosis of CS3. In addition, activity profiles of regulons associated with chromatin remodelling highlighted additional potential regulatory differences among three colorectal cancer subtypes, indicating that epigenetically driven transcriptional networks might be important differentiators of these molecular subtypes (**Figures 6E** **,** **F**).

**Figure 7:**
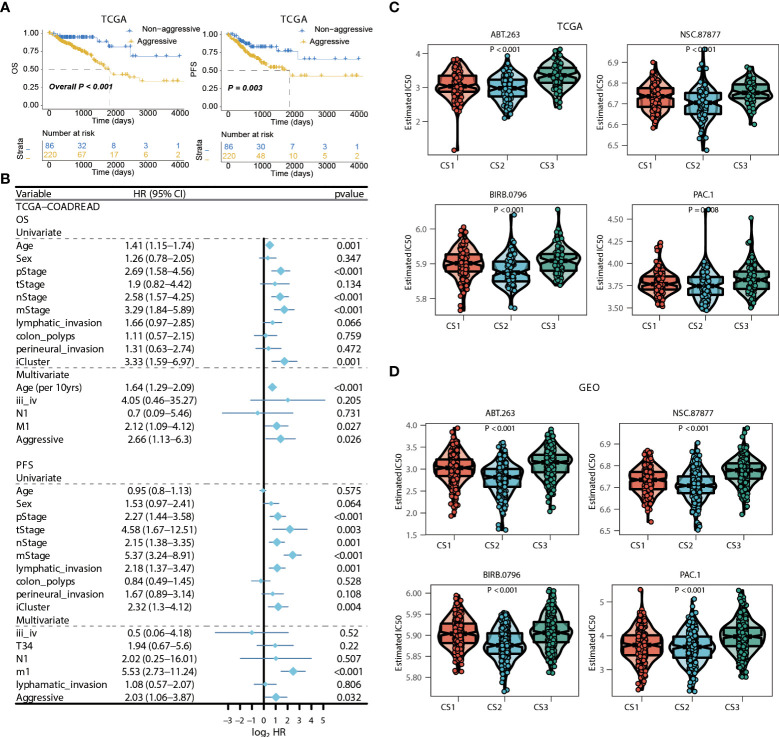
Identification of transcriptome biomarkers for colorectal cancer subtype. **(A)** KM of os using movics agreesiveness in coadread of tcga. **(B)** Forest plot showing the hazard ratio (95% CI) in univariate and multivariate Cox regressions with the corresponding *P* values. **(C, D)** Boxplot showing the distribution of estimated IC50 among three cancer subtypes based on GDSC database, **(C)** TCGA, **(D)** GEO.

### Independent prognostic value of colorectal cancer subtype

We then surveyed that whether the current classification was an independent prognostic factor in colorectal cancers from TCGA cohort. As the generally favourable prognosis of CS3, we therefore considered the CS3 as the non-aggressive subtype while patients belonged to CS1 or CS2 were aggressive in clinical setting. In this manner, univariate Cox regression model was first conducted to filter out prognostic clinical characterizations concerning OS and PFS; multivariate Cox regression was subsequently performed based on those prognosis-relevant features. Using such strategy, we found that the current classification remained the independent prognostic factor after adjusting clinical prognostic features with respect to OS (*P* = 0.026) and PFS (*P* = 0.032) (**Figures 7A, B**).

### Potential therapeutic strategy for colorectal cancer subtype

Considering the significantly poor clinical outcome of CS2 in colorectal cancer, we decided to infer potential anticancer agents that may show clinical efficiency for patients belonging to CS2 through an *in-sillico* drug screening approach. To this end, we constructed ridge regression model between cell lines and corresponding drug sensitivity and applied the predictive model to each of the colorectal cases in both TCGA and GEO cohorts (**Supplementary Tables 12, 13**). A total of four drugs were discovered to be potentially effective in treating patients with CS2 phenotype as compared to other cases, including ABT.263, NSC.87877, BIRB.0796, and PAC.1 (all, *P* < 0.01; **Figures 7C** **,** **D**).

### Identified a biomarker for multi-omics molecular subtype

**Figure 8:**
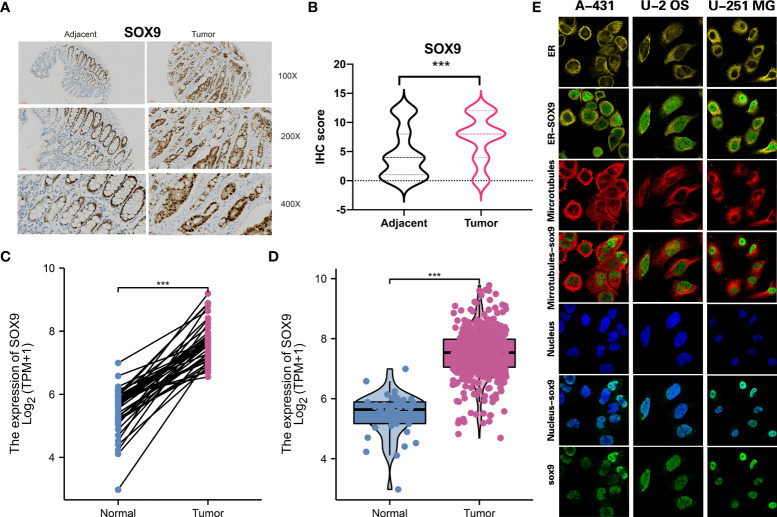
Molecular subtype biomarkers validated by wet experiment. **(A)** SOX9 protein expression of the represent sample in adjacent and tumor samples by IHC. **(B)** Pair-test for SOX9 protein expression between 50 tumor tissue and 50 tumor adjacent tissue by IHC. **(C)** and **(D)** SOX9 gene expression between tumor tissue and adjacent tissue in TCGA-COAD public cohort. **(E)** The location of SOX9 in A-431, U-2 OS and U-251 MG in the Human Protein Atlas(HPA). ***p<0.001.

To apply the molecular subtype better in the clinic, we identified a biomarker for our molecular subtype which most based on the DNA mutation and RNA expression among different subtypes. By using the Chi-square test for DNA mutation and fold change with adjust FDR value for RNA expression, then, we detected SOX9 was a significant gene in the CS3 subtype. Through the IHC experiment, we found SOX9 was higher in the 50 tumor tissue than the 50 tumor adjacent tissue. IHC showed the represent sample in adjacent and tumor samples (**Figures 8A, B**). TCGA-COAD public cohort also confirmed that SOX9 was higher in tumor tissue than that in adjacent tissue (**Figures 8C, D**). SOX9 mainly located in the nucleoplasm of cell in A-431, U-2 OS and U-251 MG multi cell lines by immunofluorescence with HPA001758 antibody in the Human Protein Atlas(HPA) **(** **Figure 8E**).

## Discussion

The high incidence and mortality of CRC have brought a huge burden on patients. How to effectively judge the prognosis of CRC patients and correctly evaluate the severity of the disease of CRC patients are the main objectives of the study on the prognosis of CRC. The prognosis of patients based on traditional tumor typing is often very different. Molecular typing of tumors can better reflect the differences in internal molecular characteristics of tumors, which is the basis for the realization of precision medicine. Accurate identification of patients’ molecular subtypes will help to accurately predict patient prognosis and develop personalized treatment plans.

Currently, tumor molecular subtype studies are mainly based on single omics data, such as transcriptomics, proteomics, genomics, etc (56–61). Bhattacharjee et al. divided lung adenocarcinoma into 4 subtypes by analyzing gene expression profile data from lung adenocarcinoma patients, and found that abnormal expression profile can be used to distinguish primary and metastatic adenocarcinoma of lung (58). Based on genomic CNV data, Shibata et al. divided lung adenocarcinoma into three subtypes by unsupervised clustering analysis, and found that patients with EGFR mutations had shorter disease-free survival times (60). As for CRC, Roepman et al. conducted unsupervised classification of genome-wide data of CRC patients based on EMT, microsatellite instability caused by mismatch repair gene defects, and high mutation frequency associated with cell proliferation (62). Meanwhile, Lai et al. proposed the co-ordinate immune response cluster (CIRC), and identified four patient groups by this method (63). Zhang et al. identified two molecular subtypes, C1 and C2, based on cell cycle-related genes. PIK3CA, RYR2 and FBXW7 mutations were more frequent in C1, and the clinical characteristics and prognosis of patients were relatively poor (64). In addition to the above genotyping based on gene mutations and cytogenetic changes in the genome (10, 21, 65–68), CRC was also classified based on differences in gene expression profiles and proteomic (69–72)biomarkers. Therefore, molecular typing based on omics data can effectively identify clinically relevant tumor subtypes, which plays a very important role in judging patient prognosis and guiding clinical treatment.

Nevertheless, any single omics data can only reflect the intrinsic molecular characteristics of tumors from a single perspective, and the contribution of single-omics analysis to tumor typing is one-sided. Therefore, the integration of multi-omics information can simultaneously capture the heterogeneity of tumors in different omics and integrate the information from multiple perspectives to identify more accurate tumor molecular typing. As the high heterogeneity of tumors is determined by multiple omics, such as genome, epigenome, transcriptome, and proteome, the analysis of data from different omics sources is expected to better reveal the mechanism of tumor genesis and development. For the first time, Matan Hofree et al. integrated genomic mutations and protein interaction networks for molecular typing of tumors to identify subtypes significantly associated with clinical features (73). Ronglai Shen et al. integrated genomic mutations, CNV and transcriptome expression profiles to obtain tumor classification based on iCluster (74). Herein, using transcriptome, DNA methylation, and driver mutations of CRC, we developed a classifier based on multi-omics integration for the prognosis prediction of CRC for the first time. At present, many studies have proved that CRC is the result of accumulation of multiple gene mutations and epigenetic modifications, and DNA hypermethylation or hypomethylation can be used as epigenetic biomarkers to predict the occurrence and prognostic effects of CRC (75–77). Driver mutations in the genome can be viewed as responsible for molecular changes associated with CRC progression, so targeting such genes for the elimination of multiple CRC gene dependencies could significantly improve efficacy (78). In conclusion, CRC can be comprehensively understood from multiple omics based on transcriptome, DNA methylation, and driver mutation levels to predict prognosis and guide clinical medication.

In the medical field, prognostic models need to undergo extensive and rigorous validation before they can be used in practice, and they also need to be continuously evaluated by feedback. At present, due to the different data standards and coding systems used by different sources, the output platforms and schemes of omics data also have certain heterogeneity. Therefore, the current integrated prognostic models are often internally verified by resampling or cross-validation. The few externally validated integrated prognostic models often involve only one type of omics data and have been externally validated in only a few open data sets, making it difficult for the current integrated prognostic models to be applied in clinical practice. In order to verify the reproducibility of the colorectal molecular subtypes we identified, we combined four external datasets from GEO cohort. We removed batch effects across different datasets and predicted the identified molecular subtypes in the GEO cohort using NTP algorithm. CS3 presented with the most favourable clinical outcome out of the three subtypes, indicating the accuracy of the subtype system.

Beyond that, there are several new findings and notable advantages to our study. TME and tumor cells interact and co-evolve to drive tumor growth and progression, and also play an important role in regulating tumor sensitivity to treatment (79). The results showed that CS1 was highly immune-infiltrated, CS3 showed relatively higher immunocyte infiltration, while CS2 was generally immune-depleted, explaining the difference in prognosis. Immunotherapy is an important treatment for CRC. We compared the responses of the three subtypes to immune checkpoint inhibitors. Abnormal metabolism is closely related to the occurrence, development, recurrence, metastasis, and prognosis of CRC. We found that the enrichment level of carbohydrate, amino acid, and lipid metabolism-relevant pathways in CS3 was higher. Our results showed that CS2 tended to loss methylations while CS3 tended to gain methylations. ABT.263 is a small molecule Bcl-2 inhibitor that can induce cell apoptosis (80). BIRB.0796 is one of the most potent compounds of (81) p38 inhibitors. PAC.1 (Caspase activator) is an effective procaspase-3 activator, which acts on primary cancer cells and induces apoptosis (82). Our findings showed that ABT.263, NSC.87877, BIRB.0796, and PAC.1 were discovered to be potentially effective in treating patients with CS2 phenotype.

Nonetheless, some limitations of the current study should not be ignored. Hence, the cases of CRC patients were relatively small; more cases are needed to confirm our conclusions. The molecular subtypes of CRC were based on retrospective cohorts. Therefore, prospective studies are needed in the future. Even though we developed molecular subtypes based on integrated multi-omics, the metabolomics and proteomics data were missing because the relevant omics information was not available in the TCGA database. With the development of information technology and genetic testing technology, more and more clinical data in the form of accessible electronic medical records and shared omics data are available. The rapid development of artificial intelligence technology can further mine the correlation and interaction between different scales of data and more effectively use different scales of data for information complementarity to achieve a more accurate prediction model. Therefore, it is of great significance to further improve and optimize the multi-omics analysis based on this study, realize the multi-center collaborative multi-omics integrated analysis, and apply it to the prognostic analysis of CRC.

## Conclusion

Taken together, we carried out multi-omics analysis of transcriptome mRNA expression, DNA methylation, and colorectal cancerous driver mutations. Three molecular subtypes were constructed and clinical significances, such as prognosis, mechanisms, and clinical therapeutic targets were observed among them. Besides, the subtypes were independent prognostic factors.

## Data availability statement

The datasets presented in this study can be found in online repositories. The names of the repository/repositories and accession number(s) can be found in the article/**Supplementary Material**.

## Author contributions

CZ, TX, LC, XZ, XL, KC, and XC designed the study. All authors contributed to the article and approved the submitted version.

## Funding

This work was supported by the Sponsored by Longyan City Science and Technology Plan Project (Grant number: 2020LYF17029).

## Conflict of interest

The authors declare that the research was conducted in the absence of any commercial or financial relationships that could be construed as a potential conflict of interest.

## Publisher’s note

All claims expressed in this article are solely those of the authors and do not necessarily represent those of their affiliated organizations, or those of the publisher, the editors and the reviewers. Any product that may be evaluated in this article, or claim that may be made by its manufacturer, is not guaranteed or endorsed by the publisher.

## Abbreviations

CRC, Colorectal Cancer; TME, Tumor Microenvironment; TMB, Tumor Mutation Burden; OS, Overall Survival; MSI, Microsatellite Instability; MMR, Mismatch Repair; SNV, Single Nucleotide Variation; TCGA, The Cancer Genome Atlas; COAD, Colon Adenocarcinoma; READ, Rectum Adenocarcinoma; FPKM, Fragments Per Kilobase Million; CNV, Copy Number Variation; RMA, Robust Multichip Average; TPM, Transcripts Per Kilobase Million; PCA, Principal Component Analysis; CPI, Clustering Prediction Index; GSVA, Gene Set Variation Analysis; MeTIL, Methylation of Tumour-Infiltrating Lymphocyte; GSEA, Gene Set Enrichment Analysis; ssGSEA, single-sample GSEA; MSigDB, Molecular Signatures Database; DMP, Differentially Methylated Probe; DPI, Data Processing Inequality; CS, Cancer Subtype; PFS, Progression-Free Survival; CIRC, Co-ordinate Immune Response Cluster.
