# Supplementary figures and images for: Immune depletion of the methylated phenotype of colon cancer is closely related to resistance to immune checkpoint inhibitors
Source: Front Immunol. 2022 Sep 8;13:983636. doi: 10.3389/fimmu.2022.983636 (PMC9492852; doi:10.3389/fimmu.2022.983636)

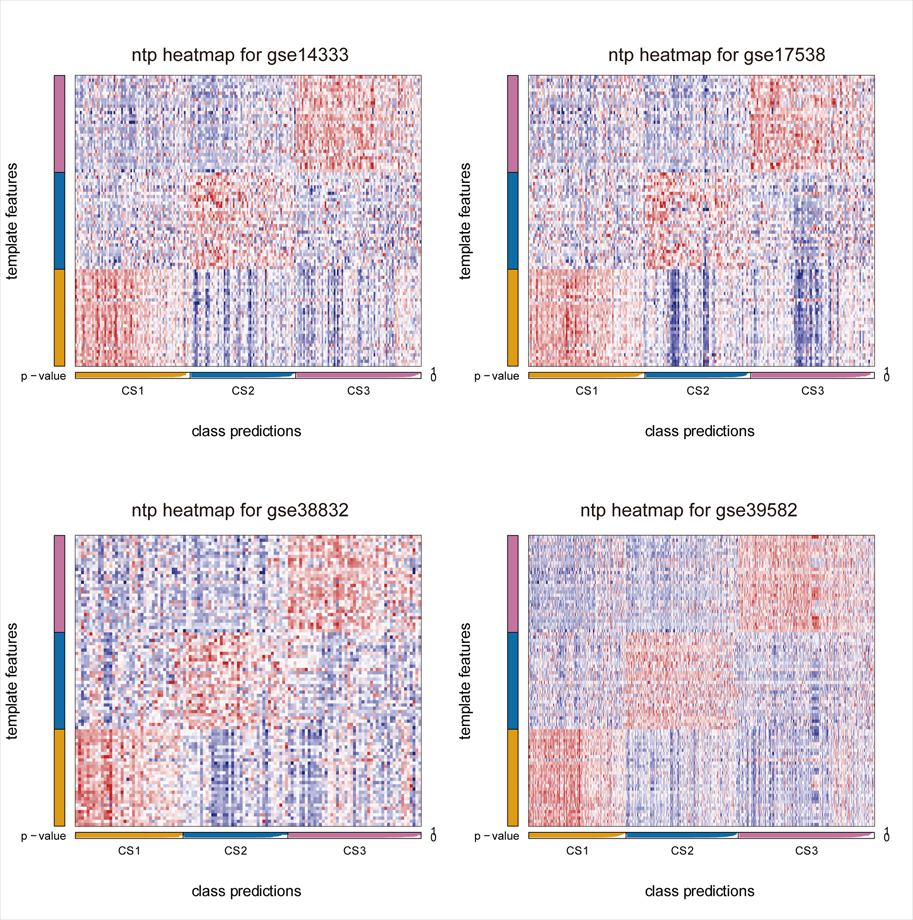

Supplement: Supplementary file 2 [file Image_1.tif]

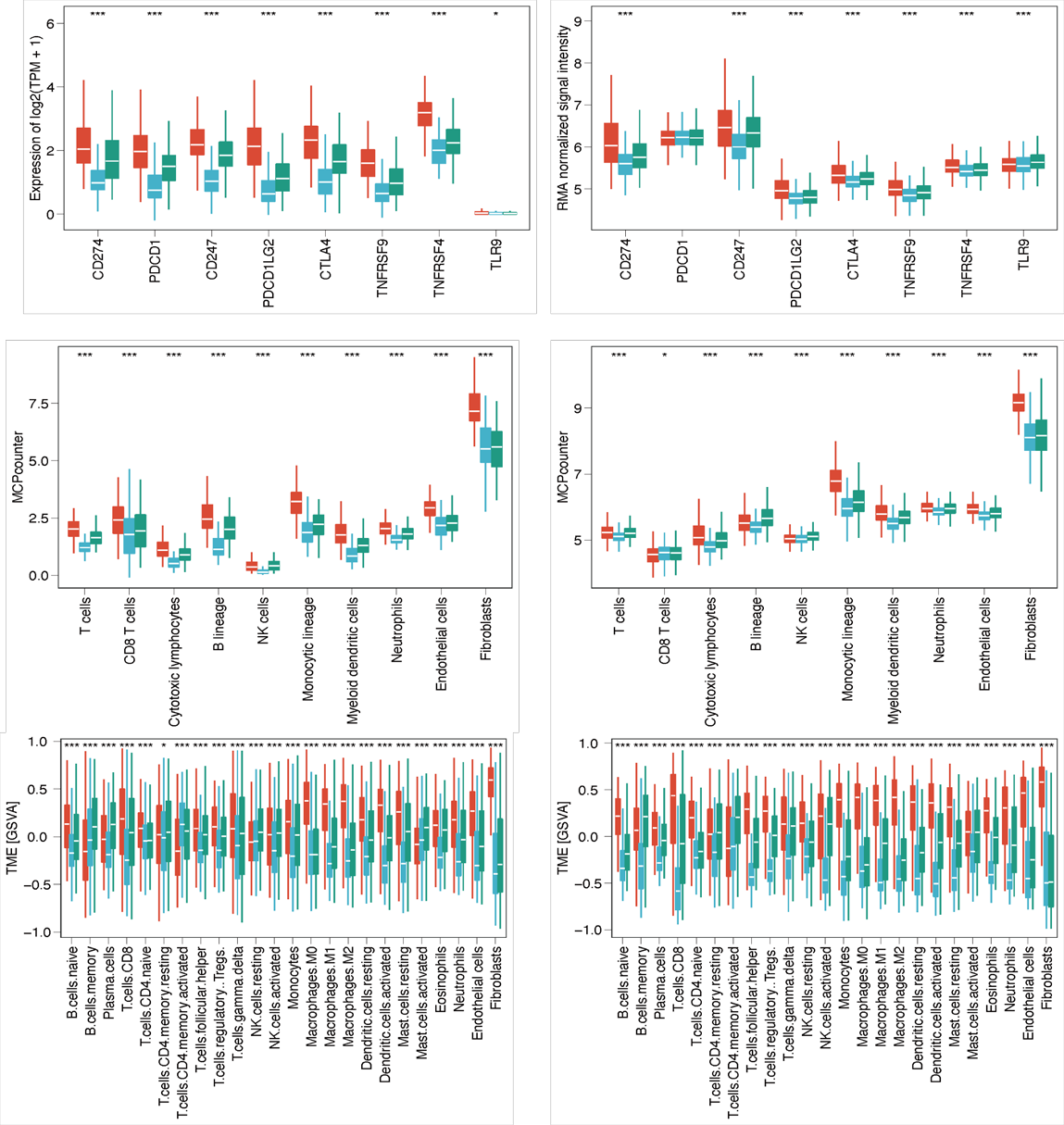

Supplement: Supplementary file 3 [file Image_2.tif]
